# Identification of high-risk factors for prehospital delay for patients with stroke using the risk matrix methods

**DOI:** 10.3389/fpubh.2022.858926

**Published:** 2022-11-11

**Authors:** Zihan Gao, Qinqin Liu, Li Yang, Xuemei Zhu

**Affiliations:** ^1^School of Nursing, Qingdao University, Qingdao, China; ^2^School of Nursing, Peking University, Beijing, China; ^3^School of Nursing, Harbin Medical University, Heilongjiang, China

**Keywords:** acute ischemic stroke, prehospital delay, risk assessment, risk matrix, Borda count

## Abstract

**Background:**

Stroke has become a leading cause of mortality and adult disability in China. The key to treating acute ischemic stroke (AIS) is to open the obstructed blood vessels as soon as possible and save the ischemic penumbra. However, the thrombolytic rate in China is only 2.5%. Research has been devoted to investigating the causes of prehospital delay, but the exact controllable risk factors for prehospital delay remain uncertain, and a consensus is lacking. We aimed to develop a risk assessment tool to identify the most critical risk factors for prehospital delay for AIS patients.

**Methods:**

From November 2018 to July 2019, 450 patients with AIS were recruited. Both qualitative and quantitative data were collected. The Delphi technique was used to obtain expert opinions about the importance of the risk indices in two rounds of Delphi consultation. Then, we used the risk matrix to identify high-risk factors for prehospital delay for AIS patients.

**Results:**

The risk matrix identified the following five critical risk factors that account for prehospital delay after AIS: living in a rural area; no bystanders when stroke occurs; patients and their families lacking an understanding of the urgency of stroke treatment; patients and their families not knowing that stroke requires thrombolysis or that there is a thrombolysis time window; and the patient self-medicating, unaware of the seriousness of the symptoms, and waiting for spontaneous remission.

**Conclusions:**

The risk analysis tool used during this study may help prevent prehospital delays for patients with AIS.

## Introduction

Stroke is becoming a significant health burden and one of the leading causes of death worldwide (1). Acute ischemic stroke (AIS), which is the most common type of stroke, accounts for approximately 73–87% of all strokes (2). Evidence indicates that the administration of recombinant tissue-type plasminogen activator (t-PA) is an effective intervention for early treatment of AIS (3). Although t-PA has been approved for stroke treatment for more than 20 years, only 11.1% of patients in developed countries (4) and 2.5% in China (5) eventually receive thrombolytic therapy.

Prehospital delay is determined by when the time from the onset of the incidence until the patient's arrival exceeds 3 h (6). Prehospital delay is the main cause of low thrombolysis rate in patients with AIS (7). Therefore, effective measures are to be taken by healthcare professionals to reduce prehospital delay.

Many factors can contribute to the prehospital delay of patients with stroke, including demographic (e.g., age, sex, education, income), clinical (e.g., symptom etiology, symptoms, clinical history, and timing of symptom onset), and cognitive-behavioral (e.g., symptom recognition and perceived severity) factors (6, 8–10). Various risk factors have been suggested, but controversy persists. Some scholars believed that age does not affect prehospital delay (11–13), whereas others have an opposite opinion. One study have found that prehospital delay is increased for elderly patients (14), whereas other studies have found that elderly patients tend to focus more attention on their condition, thereby reducing the incidence of prehospital delay (15, 16). It has been suggested that the level of education does not affect prehospital delay (11). However, some have argued that the level of education would directly affect the cognition of stroke patients with risk awareness. Therefore, the incidence of prehospital delay is reduced for individuals with higher educational levels (17). In addition, patients with a history of diabetes may have symptoms that mimic autonomic nervous disorders after hypoglycemia, such as limb weakness, unclear speech, sensory disorders, dizziness, and confusion, and other pre-stroke symptoms, which may be misdiagnosed, thus, resulting in stroke prehospital delay (9). Studies have shown that some patients with diseases that require self-management (such as atrial fibrillation) are more likely to detect abnormalities and be more aware of their condition, and less likely to have a delayed response to symptoms (18). Additionally, studies have not yet reached a unified conclusion regarding the role of the timing of symptom onset in prehospital delay. One study pointed out that during the daytime, patients are conscious and can identify their abnormalities immediately (12). Accordingly, during the night, when patients are asleep, the detection of the onset of symptoms is relatively more likely to be delayed. However, some scholars have had different opinions; for example, one study indicated that because there is less traffic at night, patients can travel to the hospital faster and receive timely treatment after symptom onset (19). Therefore, prehospital delay for stroke patients depends on many controversial factors that can occur at any stage before the arrival of the patient at the hospital (20). Research has been devoted to investigating the causes of prehospital delay, but the exact controllable risk factors for prehospital delay remain uncertain, and a consensus is lacking. One key barrier to prehospital delay prevention is the lack of risk assessment tools to evaluate existing or potential prehospital delay risks. The availability of a highly accurate risk assessment tool would facilitate proactive identification of individuals in need of more intensive monitoring and proper intervention. Therefore, risk management approaches can be used to address this problem.

Prehospital delay risk management is not only the management of a single risk factor of prehospital delay in the past, but also the integrated management of all risks from the perspective of the whole system. This study systematically sorted out and screened various risk factors leading to stroke prehospital delay from the levels of patients, their families, and emergency systems, and identified the key risk factors leading to stroke prehospital delay. Identifying the hierarchical importance of risk factors for prehospital delay may help healthcare professionals effectively identify people at high risk and provide appropriate interventions. This method can prevent prehospital delay and allow for efficient utilization of healthcare resources.

The objective of this study was to develop a risk assessment tool to estimate and stratify the critical risk factors for prehospital delay. A univariate analysis was used to obtain important risk indicators of prehospital delay for stroke patients. The Delphi method was utilized to obtain experts' opinions regarding scoring the impact and occurrence possibilities of these risk indices to quantify agreement among experts regarding their level of importance. After two rounds of expert consultation, a risk matrix was constructed according to the quantitative results of each risk factor. The Borda count method was used to calculate the weight allocated to each of the final risk indices. The risk matrix method combined with the Borda count method comprehensively evaluates risk classes and avoids risk ties.

## Methods

### Sample size calculation

The sample size was calculated using a sample size formula for a descriptive cross-sectional study: *n* = *Z*α^2^*p* (1 – *p*)/*d*^2^, where *n* is the minimum desired sample size, *Z*α is the standard normal deviation (usually set as 1.96) that corresponds to a 5% level of significance, *p* is the prevalence, and *d* is the degree of accuracy (precision). Previous studies have shown that the incidence of prehospital delay for stroke patients was 60% (21); therefore, *d* was set at 5%, and *n* = 1.96^2^ × 0.6 × (1 – 0.6)/0.05^2^ = 369. After anticipating an approximate drop-out rate of 15% during the study course, 450 participants were recruited.

### Data collection

This study was approved by a local Institutional Ethics Committee. We included patients who were 18 years or older, admitted to a tertiary hospital between November 1, 2018 and July 31, 2019, had AIS diagnosed by clinical examination and neuroimaging results (at least one computed tomography or magnetic resonance imaging brain scan), and were willing to participate in the present study. The exclusion criteria were patients with transient ischemic attack or cerebral hemorrhage, cognitive impairment, or inability to answer questions. We also excluded patients whose time from disease onset to admission could not be determined.

### Variable measurements

#### Prehospital delay risk factors

We identified the risk factors for prehospital delay in stroke patients through literature review and qualitative interviews. Based on the review of current evidence, we obtained 59 risk factors related to stroke prehospital delay. In addition, we conducted face-to-face interviews on risk factors of stroke prehospital delay with four patients and their families, two community general practitioners, one emergency physician, eight neurologists, and four emergency center staffs. The content analysis method was used to analyze the interview data, and a total of 35 risk factors were extracted. After excluding repeated risk factors among the results of literature review and qualitative interviews, a total of 64 risk factors were left as the basis for the formulation of the questionnaire. Therefore, the self-designed questionnaire had five sections with 64 items comprising sociodemographic factors, clinical factors, patients' medical decisions, transport factors, and time records. Socioeconomic factors included age, sex, educational level, family income, and type of medical insurance. Clinical characteristics included clinical history, primary symptoms, stroke severity, stroke type, and timing of symptoms. Patients' cognitive-behavioral factors included stroke knowledge, identification of premonitory symptoms, and assessment of symptom severity. Transport factors included the use of emergency medical services and transfer experience. Time records included the time when stroke symptoms were detected, the time when it was decided that medical attention was necessary, and the time of arrival at the hospital.

#### Identification of aura symptoms

Aura symptoms were identified and assessed using an alertness questionnaire regarding premonitory symptoms of stroke that was developed by Zhang et al. (22). The questionnaire comprises nine items and uses a two-point system: a score of 1 is given for a correct answer and a score of 0 is given for a wrong answer.

#### Stroke knowledge

The Stroke Knowledge Scale was used to evaluate stroke knowledge. The scale was developed in 2016 by Yao (23) and includes six dimensions (stroke symptoms, first aid measures, risk factors, safe drug use, health behavior, and rehabilitation knowledge). A total of 40 items are scored using a two-point system: a score of 1 is given for the correct answer and a score of 0 is given for the wrong answer or no answer. The total score ranges from 0 to 40.

### Delphi consultations

In this study, the selection criteria of the experts were as follows: (a) engaged in stroke medical work; (b) had 5 years or more experience in stroke medical work; and (c) able to understand the content of the questionnaire and implications of the relevant indexes. The questionnaire was sent to the experts by e-mail after receiving their prior approval. The responses of each participant remained anonymous throughout each survey round and were analyzed anonymously. Only team members knew the identifiable responses from each participant. Delphi participants did not know the identities of the other Delphi participants during the Delphi study. Experts then defined the basis of their judgment according to whether their view was based on theoretical knowledge, practical experience, learned from peers, or intuitive feeling (24).

Eighteen experts participated in the consultations; 33.33% were males (6/18) and 66.67% were females (12/18). All experts responded to the two rounds of Delphi questionnaires. They were distributed in Heilongjiang Province, Shandong Province, and Henan Province. Their average age was 42.8 years (range, 32–50 years). Their average professional experience was 15.7 years (range, 6–27 years). Regarding the academic level of the experts, 33.3% (6/18) were professors, 61.1% (11/18) were associate professors, and 5.6% (1/18) were attending physicians. Their major areas of expertise were emergency medicine (1/18) and neurology (17/18). During the two rounds of Delphi consultation, 18 questionnaires were issued and recovered, with a 100% recovery rate.

### Data analysis

SPSS software was used for data entry and computation. Missing values were also obtained using the maximum likelihood method in SPSS. Responses to the survey questions were analyzed using descriptive statistics (significance was set at *P* < 0.05). Using the prehospital delay time (3 h) as a cut-off point, patients were divided into two groups: the timely admission group (≤ 3 h) and the delayed admission group (>3 h). Qualitative variables were expressed as numbers (percentages). The chi-square test was used for comparisons. Continuous normally distributed variables were expressed as mean ± standard deviation and compared using Student's *t*-test.

#### Risk level assessment method

A risk matrix was constructed based on the identified risk factors for prehospital delay combined with expert opinions. The Borda count is designed to rank different risk factors according to their importance. The risk matrix is a structured approach used to identify the importance of risk during project management. It assesses the potential impact of risk through a simple operational method and qualitative and quantitative analyses. The traditional risk matrix level is determined by the combination of the probability of occurrence of the risk and the severity of the consequences. Risk grading according to the corresponding values was presented in Table 1.

**Table 1:** Risk rating scale.

**Probability of risk occurrence**	**Severity of the consequences**				
	**Negligible**	**Minor**	**Moderate**	**Severe**	**Critical**
Very unlikely	I	I	II	III	III
Less likely	I	II	II	III	IV
Likely	II	II	III	IV	IV
More likely	III	III	III	IV	V
Very likely	III	IV	IV	V	V

The Borda count method is a well-known social choice method that is frequently used for group decision-making problems (25). This method can determine the winner of an election by giving each candidate a certain number of points corresponding to the position at which each voter ranks them. Risk factors are sorted based on the significance of the following criteria that reduce the subjectivity of experts. *k* is the different risk assessment criteria (*k1, k2*, and *k3* represent the risk impact, risk probability, and risk level, respectively). *N* is the total number of risks; *i* is the total number of risks for risk *i* (1 ≤ *i* ≤ *N*); *R*_*ik*_ represents the *k* criterion, which is the risk level of the number *i* ranked, that is, the number of risks that are more severe than risk *i* among the total risks of *N*. The Borda number determines the Borda count. A Borda count of 0 indicates the most critical risk. The Borda number of risks *i* can be obtained by the following formula:


(1) $$\begin{array}{l} b_{i}=\underset{k=1}{\sum }\left(N-R_{ik}\right)\; \end{array}$$

The risk level is determined by the probability of risk occurrence and the severity of the consequences. During this study, the risk grade was divided into five levels: I, II, III, IV, and V. Scores ranged from I to V, with higher scores indicating higher risk rating.

## Results

### Determination of 28 risk evaluation metrics

A total of 450 patients admitted to the neurology ward were recruited and enrolled in the study. Only 12.7% of the patients arrived at the hospital within 3 h, and 32.4% of the patients had never known about or heard of stroke. Significant factors in the univariate analysis (*P* < 0.05) were applied in the risk indicator system for prehospital delay (Table 2). Twenty-eight specific risk indicators were identified, including patient delay and transport delay risk (Table 3).

**Table 2:** Prehospital delay according to patient characteristics (*n* = 450).

**Factors**	**Prehospital delay time < 3 h (*n* = 57)**	**Prehospital delay time ≥3 h (*n* = 393)**	***P-*value^a^**
Having commercial medical insurance, No. (%)	12 (21.05)	44 (11.20)	0.035^b^
Physical examination status ≥1/year, No. (%)	23 (40.35)	90 (22.90)	0.005^b^
Living in Urban, No. (%)	51 (89.47)	247 (62.85)	< 0.001^b^
Family history of stroke, No. (%)	21 (36.84)	94 (23.92)	0.037^b^
Wake-up stroke, No. (%)	0 (0.00)	82 (20.87)	0.007^b^
Symptom onset suddenly, No. (%)	51 (89.47)	262 (66.67)	< 0.001^b^
Language impairment, No. (%)	37 (64.91)	191 (48.60)	0.021^b^
Unilateral facial numbness or weakness, No. (%)	23 (40.35)	99 (25.19)	0.016^b^
Bilateral facial numbness or weakness, No. (%)	3 (5.26)	67 (17.04)	0.022^b^
Left arm weakness or numbness, No. (%)	27 (47.37)	131 (33.33)	0.038^b^
Unconsciousness or fainting, No. (%)	3 (5.26)	2 (0.01)	0.016^b^
Dizziness, loss of balance, difficulty walking, or loss of coordination, No. (%)	21 (36.84)	203 (51.65)	0.037^b^
Considered any of the symptoms to be severe, No. (%)	46 (80.70)	150 (12.72)	< 0.001^b^
Recognized the problem as stroke, No. (%)	36 (63.16)	115 (29.26)	< 0.001^b^
Bystanders' reactions suggested going to a hospital, No. (%)	56 (98.24)	272 (69.21)	< 0.001^b^
Referred from other hospitals, No. (%)	6 (10.53)	127 (32.32)	0.020^b^
Used an ambulance for this occurrence, No. (%)	13 (22.80)	18 (4.58)	< 0.001^b^
Symptom change before admission, No. (%)			
No change	27 (47.37)	102 (25.95)	< 0.001^b^
Exacerbated	12 (21.05)	166 (42.24)	
Lightened	12 (21.05)	15 (3.82)	
Fluctuated	6 (10.53)	110 (27.99)	
Bystanders recognized the problem as stroke, No. (%)			
Yes	39 (68.42)	130 (33.08)	< 0.001^b^
No	9 (15.79)	137 (34.86)	
Unknown	9 (15.79)	126 (32.06)	
Patient's response when symptoms first appeared, No. (%)			
Contacted relative/acquaintance	27 (47.37)	96 (24.43)	< 0.001^b^
Called a physician	1 (1.75)	1 (0.00)	
Went directly to a hospital	17 (29.82)	54 (13.74)	
Self-administered medicine	2 (3.50)	65 (16.54)	
Called for emergency assistance	6 (10.53)	3 (0.01)	
Did nothing	4 (7.01)	174 (44.27)	
Knowledge about the time window for intravenous thrombolysis for stroke, No. (%)			
≤ 3 h	13 (22.80)	19 (4.83)	< 0.001^b^
≤ 4.5 h	7 (12.28)	10 (2.54)	
≤ 6 h	14 (24.56)	46 (11.70)	
Unknown	23 (40.35)	318 (80.91)	
Monthly household income per capita (yuan), No. (%)			
< 1,000	4 (7.01)	47 (11.96)	0.005^b^
1,000–2,999	22 (38.60)	204 (51.90)	
3,000–5,000	14 (24.56)	92 (23.41)	
>5,000	17 (29.82)	50 (12.72)	
Onset location, No. (%)			
Home	36 (63.16)	270 (68.70)	< 0.001^b^
Workplace	4 (7.01)	66 (16.79)	
Public place	9 (15.79)	45 (11.45)	
In the car	6 (10.53)	4 (1.01)	
Other	2 (3.51)	8 (2.04)	
Distance between the place of onset and the investigating hospital, No. (%)			
≤ 5 km	8 (14.04)	12 (3.05)	< 0.001^b^
>5 and ≤ 10 km	9 (15.79)	32 (8.14)	
>10 and ≤ 20 km	17 (29.82)	50 (12.72)	
>20 km	23 (40.35)	299 (76.08)	
Transportation to the first hospital, No. (%)			
Car	33 (57.89)	268 (68.19)	< 0.001^b^
Ambulance	10 (17.54)	14 (3.56)	
Other	14 (24.56)	111 (28.24)	
Ischemic stroke type, No. (%)			
Anterior circulation stroke	38 (66.67)	147 (37.40)	< 0.001^b^
Posterior circulation stroke	19 (33.33)	217 (55.22)	
Unknown	0 (0.00)	49 (12.47)	
Stroke aura symptom awareness, mean (SD)^d^	7.18 (2.00)	6.58 (2.35)	0.043^c^

^a^Only statistically significant variables are included.

^b^Chi-square test.

^c^*t*-test.

^d^Stroke aura symptoms awareness questionnaire-9 is a test for stroke aura symptoms awareness.

**Table 3:** Prehospital delay risk matrix for stroke.

**No**.	**Risk factors**	**Risk impact degree**	**Quantitative value of the degree of risk impact**	**Possibility of risk occurrence**	**Quantitative value of the probability of risk occurrence**	**Risk rank**	**Borda number**	**Borda count**
A1	Living in a rural area	Critical	4.11	Very likely	4.17	V	84	0
A2	Low family income	Severe	3.67	More likely	3.83	IV	61	9
A3	No health insurance or low reimbursement	Severe	3.56	More likely	3.72	IV	61	9
A4	Physical examination less than once per year	Severe	3.11	More likely	3.22	IV	61	9
A5	Posterior circulation stroke	Severe	3.22	More likely	3.22	IV	61	9
A6	Minor symptoms (NIHSS^a^ score < 4)	Severe	3.22	More likely	3.33	IV	61	9
A7	Bilateral face cavity/mouse skew	General	2.83	Likely	2.78	III	6	26
A8	Limb weakness and numbness	Severe	3.28	More likely	3.06	IV	61	9
A9	Dizziness/unsteadiness of gait	General	2.94	Likely	3.00	III	6	26
A10	Wake-up stroke	Severe	3.50	More likely	3.33	IV	61	9
A11	Gradual progression of symptoms	Severe	3.94	More likely	3.94	IV	61	9
A12	Symptoms were alleviated prior to admission	Severe	3.50	More likely	3.56	IV	61	9
A13	No bystanders when stroke occurred	Critical	4.44	Very likely	4.06	V	84	0
A14	After stroke onset, bystanders failed to recognize the stroke	Severe	3.56	More likely	3.61	IV	61	9
A15	Patients and their families have low awareness of premonitory symptoms of stroke	Severe	3.50	More likely	3.44	IV	61	9
A16	Patients and their family members have a low level of stroke knowledge	Critical	4.17	More likely	3.94	V	79	5
A17	Attributed the symptoms of other diseases after onset	Severe	3.94	More likely	3.83	IV	61	9
A18	Patients and their families lack an understanding of the urgency of stroke treatment	Critical	4.39	Very likely	4.28	V	84	0
A19	Patients and their families do not know that stroke requires thrombolysis and that there is a thrombolysis time window	Critical	4.61	Very likely	4.33	V	84	0
A20	Patient self-medicates, does not think that the symptoms are serious, and waits for spontaneous remission	Critical	4.17	Very likely	4.11	V	84	0
B1	Patient chooses other means of transportation instead of EMS^b^	Severe	3.11	More likely	3.33	IV	61	9
B2	Onset location is distant from the general hospital	Critical	4.22	More likely	3.89	V	79	5
B3	Emergency workers lack knowledge of stroke	Severe	3.67	More likely	3.33	IV	61	9
B4	Paramedics did not contact the hospital in advance	Severe	3.61	More likely	3.39	IV	61	9
B5	Lack of first aid resources (such as insufficient ambulances, uneven medical resource distribution, blocked first aid channels, etc.)	Critical	4.11	More likely	3.78	V	79	5
B6	The hospital where the patient is treated during the first stroke is a primary medical institution and there are no corresponding treatment and thrombolysis conditions	Critical	4.22	More likely	3.83	V	79	5
B7	Traffic	Severe	3.72	More likely	3.56	IV	61	9
B8	Outside hospital transfer	Severe	3.67	More likely	3.39	IV	61	9

^a^NIHSS, National Institutes of Health Stroke Scale. ^b^EMS, emergency medical services.

### Delphi expert consultation

#### Expert authority coefficient

According to the expert self-evaluation scores, the authoritative coefficients of various indicators reached more than 0.80 for the 18 experts. The coefficient of authority was 0.95. It was demonstrated that the participating experts had a high level of familiarity with the indexes, including research and work experience in these areas. Therefore, the selection of indices and results had high credibility.

#### Degree of expert coordination

The Kendall coordination coefficient (*W*) refers to whether there are significant differences between the opinions of the experts evaluating each index. *W* ranges between 0 and 1, with a greater value indicating a higher degree of concordance between the experts. During this study, the expert coordination coefficient (*W*) was 0.254 (*P* < 0.001), which suggested that the concordance of the expert opinions was high.

### Determination of risk rating

**Figure 1:**
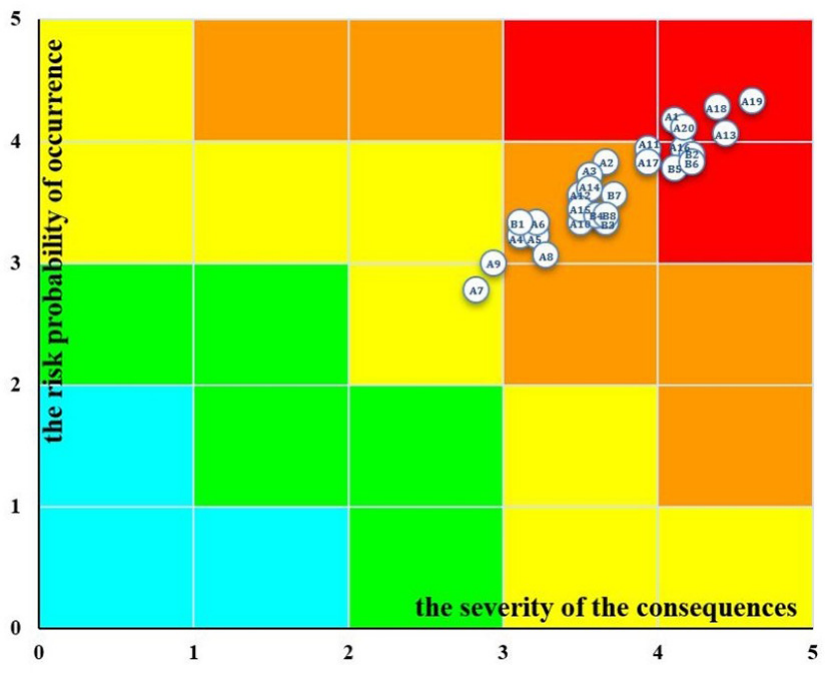
Prehospital delay risk matrix map of stroke. There are five colors in the risk matrix diagram that represent different degrees of risk and corresponding processing methods. Blue corresponds to very low risk (I), green corresponds to low risk (II), yellow corresponds to medium risk (III),orange corresponds to high risk (IV), and red corresponds to very high risk (V).

The probability of risk occurrence and the severity of consequences were used to determine the risk rating. The expert scoring methods designated the probability of risk occurrence and the severity of the consequences. All expert opinions and the mean are summarized in Table 3. The risk matrix diagram was drawn using the severity of the consequences as the abscissa and the probability of risk occurrence as the ordinate (Figure 1). The Borda scores were presented in Table 3. There were five risk factors with a Borda count of 0: living in a rural area (A1); no bystanders when stroke occurred (A13); patients and their families lacking understanding about the urgency of stroke treatment (A18); patients and their families not knowing that stroke requires thrombolysis or about the thrombolysis time window (A19); and the patient self-medicating, not thinking that the symptoms are serious, and waiting for spontaneous remission (A20). These five risk factors were the most critical for prehospital delay after stroke.

## Discussion

During the current study, 32.4% of the patients had never heard of stroke. Among patients who had heard of the disease, the single rate of awareness of common stroke symptoms and risk factors were relatively high. However, the overall awareness rates were lower compared to other studies. For example, Nordanstig et al. (26) and Sundseth et al. (27) reported rates of 50.0% and 39.5%, respectively. Our study indicated that patients have insufficient knowledge of stroke symptoms and risk factors, and their understanding of stroke prevention and treatment needs to be improved (A18, A19, and A20). Similar to previous studies (11, 28), the present study showed that prehospital delay is caused by inadequate acknowledgment of the seriousness of disease, which leads to a lack of urgency to change behaviors. Rural residence was determined as one of the critical factors for prehospital delay, which may be associated with insufficient medical resources. The survey results were consistent with the investigation results because patients with prehospital delay inhabited economically underdeveloped and geographically remote areas, and their medical and health resources were relatively scarce. Additionally, the lack of help from others at the time of stroke onset was a key factor in prehospital delay. Ischemic stroke is a heterogeneous disorder characterized by a variety of clinical symptoms. Hence, bystander awareness and intervention are essential. When a patient presents with fainting and limb stiffness or similar and/or more severe symptoms, bystanders will take immediate action. If the patient experiences mild symptoms (dizziness, numb limbs, etc.), then the delay time depends on the recognition of the severity of those symptoms. When the patients or bystanders realize something is abnormal, they should seek medical help immediately. On the contrary, if there are no bystanders, then the condition may deteriorate unless the patients are able to promptly recognize the symptoms themselves.

The results of this study can be used to develop new clinical assessment tools for the healthcare data domain. These tools may contribute to prioritizing interventions for the at-risk population and have a certain practical value. Additionally, this study creatively constructed the risk matrix and identified the key risk factors for prehospital delay with stroke, which can provide a basis for the implementation of comprehensive and effective interventions and risk prevention measures and a theoretical basis for proposing and developing management strategies.

When the five most practical factors are identified by the Borda count and risk matrix, adaptation recommendations for interventions may be proposed accordingly. Regarding the high-risk factor of living in rural areas (A1), the cost of living in rural areas may be lower, but patients in these areas are typically older and have less access to transportation. They also have lower educational levels, poorer housing conditions, higher poverty rates, poorer health, and more disabilities than their urban counterparts. In underserved rural communities, nurses are often frontline healthcare professionals. Some researchers believed that healthcare professionals implement information technology to achieve interdisciplinary medical cooperation in rural areas, which can effectively improve care; therefore, the informatics knowledge and skills of the nursing staff in rural areas must be upgraded (29). Some researchers have pointed out that telemedicine has been mainly used to access certain health services for populations living in remote areas (30). Furthermore, many researchers have pointed out that the implementation of stroke telemedicine can help increase the rate of intravenous thrombolysis, confirming that the application of stroke telemedicine to cerebrovascular diseases has excellent potential (31, 32). Therefore, strengthening the advancement of stroke telemedicine care can improve, to a certain extent, the imbalanced allocation of medical resources for stroke and the lack of senior medical personnel in remote areas.

Because one risk factor is the absence of bystanders when stroke occurs (A13), community workers should provide better care for elderly individuals living alone. Interacting with elderly people living alone through community activities, identifying their illnesses, and promptly providing appropriate care are important during the early management of stroke patients. This can be offered as part of a holistic and individualized care plan by the professional community nurse.

We attributed the following three risk factors to the lack of knowledge about stroke: A18, A19, and A20. To reduce prehospital delay for stroke patients, hospitals and community health service centers should collaborate to improve the knowledge of the general public regarding the urgency of stroke treatment, the need for thrombolysis, the thrombolysis time window, common stroke symptoms, and how to recognize the severity of stroke-related symptoms to decrease mortality and disability rates of stroke patients, especially those in rural areas. Community nurses have been patient advocates, patient educators, and case managers for quite some time. Therefore, the expertise that nurses can contribute to improving the health of underserved rural communities *via* information technology methods is considerable (29). Mass media is a powerful way of disseminating information about the health effects of prehospital delay to the general public; therefore, efforts should be made to take advantage of mass media to extend the reach of informational messages regarding stroke (28). For example, a wide range of health education activities can be regularly performed for community residents through the internet, radio, television, newspapers, publicity boards, brochures, and lectures.

This is the first study to use the risk matrix and Borda count to identify high-risk factors for prehospital delay among stroke patients. However, this study had some limitations. First, convenience samples may be biased because individuals who choose to participate in the study may not fully represent the population from which the sample has been drawn. Second, because of the cross-sectional design of the study, causality could not be inferred. Therefore, longitudinal studies are required to infer actual causal relationships.

## Conclusions

This study established a risk assessment tool to evaluate the prehospital delay risk for stroke patients using the risk matrix and Borda count. In this risk assessment framework, five significant risk factors were identified and could be used to evaluate the risk factors for prehospital delay based on the probability of risk occurrence and the severity of the consequences. This practical and objective tool can guide risk management and reduce prehospital delay activities as an essential public health strategy. It is anticipated that this prehospital delay risk assessment tool will be further developed and find further applications and will be adapted based on cultural differences. By establishing a risk assessment tool, we can continually evaluate and refine risk factors, focus on key risk factors, and perform targeted preventive measures.

## Data availability statement

The original contributions presented in the study are included in the article/supplementary materials, further inquiries can be directed to the corresponding author.

## Ethics statement

This study was approved by the Research Ethics Committee of Harbin Medical University, Harbin, China (Ref. No. KY2017-063). The study purpose statement was read by all study subjects and each provided written informed consent. The patients' identity information was kept confidential. The patients/participants provided their written informed consent to participate in this study.

## Author contributions

ZG and QL were responsible for the study conception, design, and drafting of the manuscript. ZG, LY, QL, and XZ collated data and conducted data analyses. LY was responsible for supervision. All authors made critical revisions to the manuscript.

## Funding

This work was supported by the Natural Science Foundation of Shandong province (ZR2021MG031) and the National Natural Science Foundation of China Grant (71704038).

## Conflict of interest

The authors declare that the research was conducted in the absence of any commercial or financial relationships that could be construed as a potential conflict of interest.

## Publisher's note

All claims expressed in this article are solely those of the authors and do not necessarily represent those of their affiliated organizations, or those of the publisher, the editors and the reviewers. Any product that may be evaluated in this article, or claim that may be made by its manufacturer, is not guaranteed or endorsed by the publisher.
